# Recurrent post-partum rhombencephalitis associated with anti-centromere antibody: a case report

**DOI:** 10.1186/s12883-019-1467-3

**Published:** 2019-10-14

**Authors:** Andy Jin, Jean Mamelona, Byrne Harper, Alier Marrero

**Affiliations:** 1 Dalhousie Medicine New Brunswick, 100 Tucker Parker Rd, Saint John, E2K 5E2 NB Canada; 2 Division of Neurology, Dr. Georges-L.-Dumont University Hospital Centre, 330 University Ave, Moncton, New Brunswick E1C 2Z3 Canada; 3 Division of Neurology, The Moncton Hospital, 135 MacBeath Ave, Moncton, E1C 6Z8 New Brunswick Canada; 4 Centre de Formation Médicale du Nouveau-Brunswick, Université de Sherbrooke, 100 Des Aboiteaux Street, Moncton, NB E1A 7R1 Canada

**Keywords:** Autoimmune disease, Rhombencephalitis, Anti-centromere antibody, Connective tissue disease, Case report

## Abstract

**Background:**

Rhombencephalitis (RE) is a serious condition of the brain with multiple etiologies. We report a unique case of recurrent, postpartum RE that is associated with positive anti-centromere antibody (ACA). A discussion of the case, current literature on autoimmune RE and related autoantibodies are reviewed.

**Case presentation:**

A healthy 33-year-old Caucasian patient (gravida 2, para 2) had two episodes of progressive focal neurological deficits during postpartum periods. Signs and symptoms included right-sided dysmetria, adiadochokinesia, weakness, ataxia, and photophobia. MRI revealed rhombencephalitis involving the mesencephalon of the brainstem. Extensive and comprehensive investigations using blood and cerebrospinal fluid (CSF) were consistently positive only for ACA. The first episode was successfully treated with empiric antimicrobial agents and steroid. Given the negative infectious work up with the prior episode and the nearly identical clinical presentations, the second episode was treated with corticosteroid only. This led to complete resolution of her symptoms and reversal of the brain magnetic resonance imaging (MRI) lesions.

**Conclusion:**

To the author's knowledge, this is the first report of a primary autoimmune RE during postpartum period that is associated with ACA. Immunologic causes should be considered early with any encephalitis. Given the risk of recurrence, relapse, and neurologic deterioration, regular monitoring is recommended, especially for female patients of child-bearing age. Consistent with the current literature on autoimmune RE, steroid seems to be an effective treatment for ACA-associated RE.

## Background

Rhombencephalitis (RE) is a rare inflammatory disease affecting the brainstem and cerebellum. Without prompt diagnosis and treatment, severe and life-threatening complications may ensue [1]. Multiple etiology, including infection, paraneoplastic syndrome, and autoimmune disorders, have been identified to date [1, 2]. Although less common, autoimmune RE may develop without an underlying immunologic disease or malignancy [3–5]. A primary autoimmune RE by definition is associated with specific neuronal antibodies, and a handful have been identified to date, including anti-NMDA, anti-GAD65, and anti-Hu [6]. To the best of our knowledge, there are no previous reports showing a direct link between RE and anti-centromere antibody (ACA). With an informed consent of the patient, we report a unique case of primary autoimmune RE. RE developed during postpartum periods in both of her pregnancies. The patient was consistently seropositive for ACA. Similar to the treatment of general autoimmune encephalitis [6, 7], methylprednisone proved effective for ACA-associated RE.

## Case presentation

**Fig. 1:**
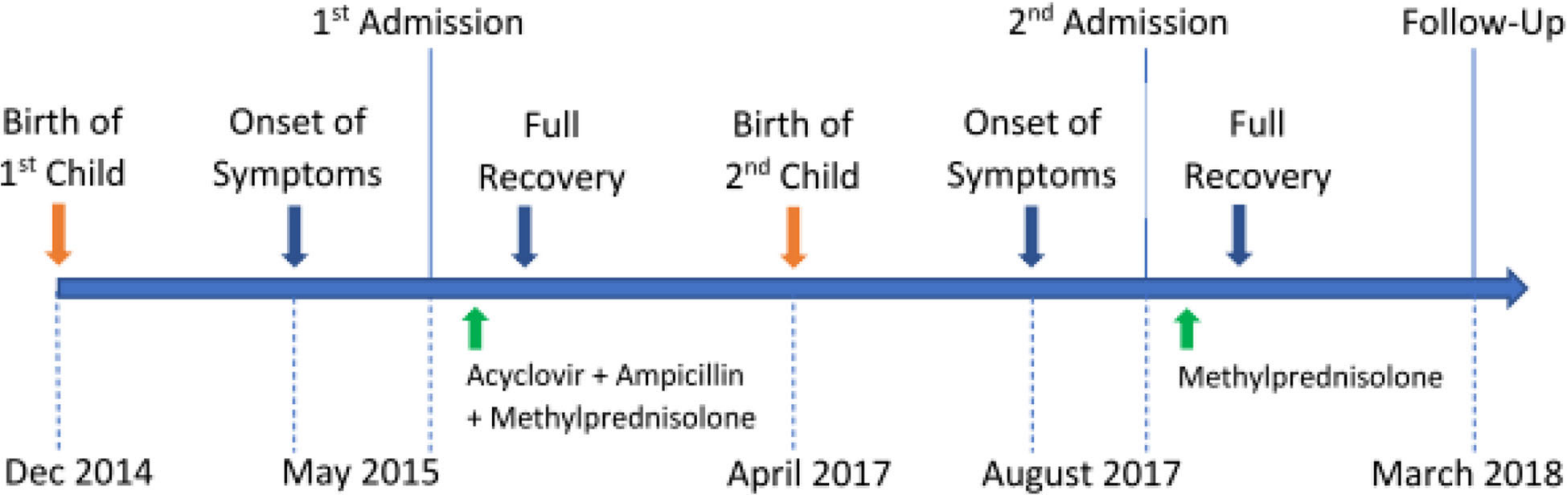
Timeline of the clinical course of the presented case. Full recovery based on clinical symptoms and repeat MRI was achieved within four months of symptoms onset

A healthy 33-year-old, right-handed Caucasian patient experienced two episodes of post-partum RE in 2015 and 2017 (Fig. 1). The first episode occurred five months after a normal vaginal delivery of her first child. She presented to the emergency department with progressively worsening right-sided dysmetria, adiadochokinesia, weakness, ataxia, and photophobia. Patient did not have any significant past medical history other than an uncomplicated infectious mononucleosis at 16 years of age. She started progestin oral contraceptives a few months after the deliveries and had no known allergies. She denied any substance use, including alcohol, tobacco, and recreational drugs. Review of systems and physical examinations were otherwise unremarkable. Vital signs, including blood pressure, were stable and within normal limits. The patient’s mother had systematic lupus erythematous (SLE).

**Fig. 2:**
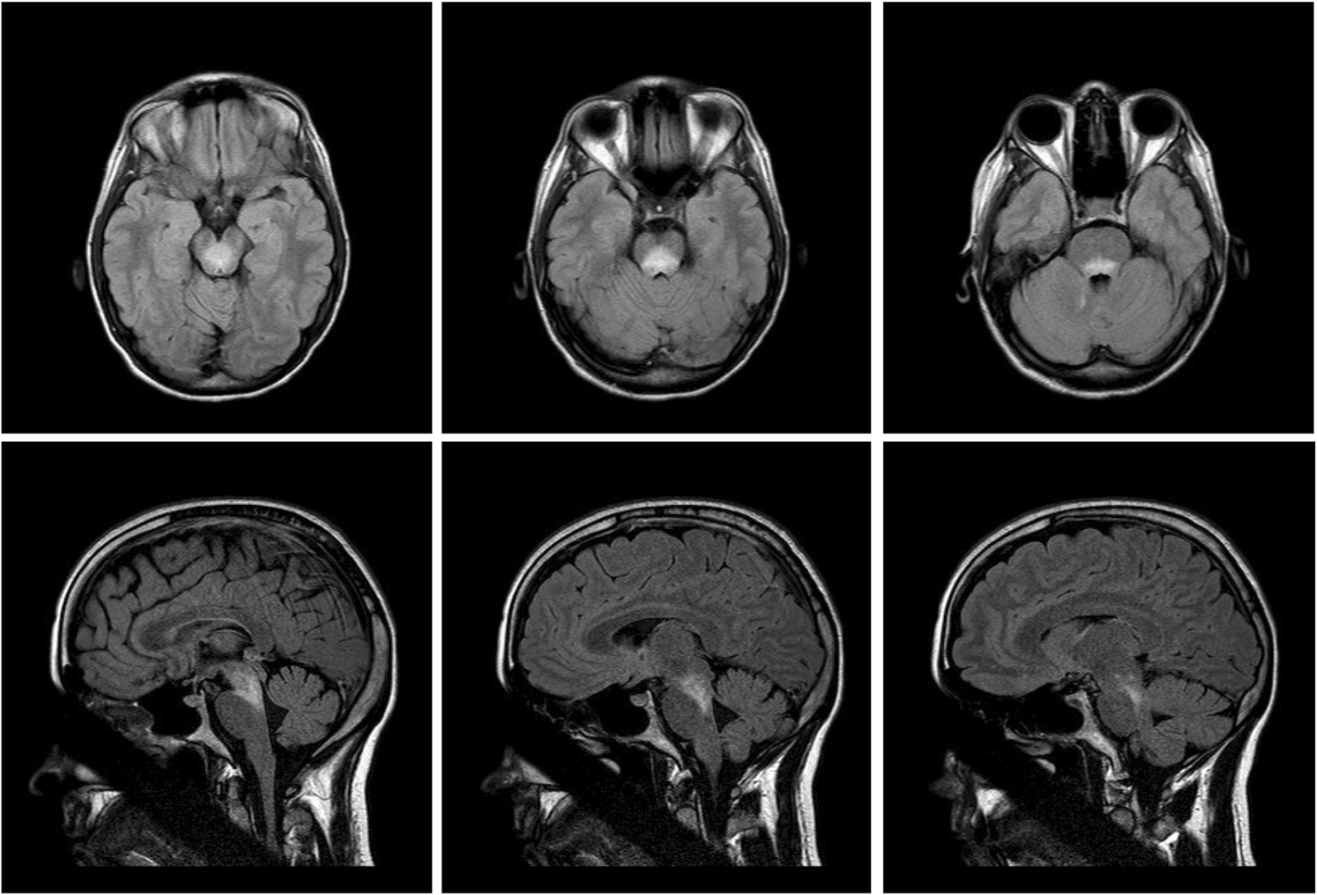
Axial (upper row) and sagittal (lower row) MRI of the brain. Gadolinium enhanced MRI with FLAIR revealed homogeneous hyperintensity involving the midbrain

Gadolinium-enhanced MRI revealed a large area of FLAIR (Fluid attenuated inversion recovery) and T2 hyperintensity that crossed the midbrain but did not involve the colliculi nor the pons, suggesting a form of RE (Fig. 2). Extensive investigations with blood and cerebrospinal fluid failed to yield any underlying etiology, such as infectious, metabolic, vascular, or demyelinating disease. Potentially abnormal findings included the following: CBC with high neutrophilia (7800 cells/μL) and leukocytosis (5000 cells/μL); CSF analysis with high albumin 53.1 mg/dl (normal: 37-51), low α1 globulin 5.4% (normal: 6.1-10.5), and low β globulin 3.9% (normal: 4.0-7.2); and viral serology with positive Epstein-Barr virus IgG and nuclear antigen, but negative IgM. CSF cell count was within normal range. With no underlying autoimmune conditions, the patient was negative for oligoclonal band and AQP-4 antibody, but the ANA screen (Table 1) came back positive for ACA.

**Table 1:** Anti-nuclear antibody (ANA) screen results

Antibody	Value (U/ml)	Cut-off value	Result
SSA/Ro	0.3	< 7	Negative
SSB/La	n.d.	< 7	Negative
Sm	3	< 7	Negative
RNP	0.6	< 5	Negative
Scl 70	0.9	< 7	Negative
JO 1	n.d.	< 7	Negative
Centromere B	212	> 7	Positive
Histone	Not available^a^	< 7	Negative
dsDNA	8.0	< 10	Negative

n.d. not detected;

^a^the exact value not found at the time we collected data

Viral and atypical infections tested were *Human immunodeficiency virus, Enterovirus, Herpes simplex, Cytomegalovirus, Varicella Zoster, Powassan virus, Eastern equine encephalitis, Jamestown Canyon virus, arbovirus, West Nile virus, Toxoplasma, Mycobacterium tuberculosis, Treponema pallidum, Listeria monocytogenes, and Borrelia burgdorferi*. Autoimmune screening included oligoclonal band, anti-NMO antibodies, cryoglobulins, complement levels, thyroid antibodies, and other autoimmune antibody panels associated with encephalitis and paraneoplastic syndrome. Conditions, including collagen disease, vasculitis, stroke, Behçet, and Hashimoto encephalopathy, could not be diagnosed with established criteria. Based on these findings and clinical symptoms, RE was diagnosed. Without a clear etiology, the patient was treated empirically with parenteral acyclovir 600 mg, ampicillin 2 g, and methylprednisolone 1 g. Although ACA remained positive post-treatment, complete resolution of symptoms and reversal of radiologic abnormalities were achieved.

Four months after the delivery of a second child, the patient presented to the ER with an acute onset of right-sided dysmetria, weakness, ataxia, photophobia, and blurred vision on the left eye. She was admitted to the hospital and seen by neurology. Physical exam revealed a slight relative afferent pupillary defect on the left eye and mild blurring of the optic disk bilaterally with normal visual field and extra-ocular movements. Vital signs were normal. Comprehensive investigations, including cytology and microbiology, were conducted. Whipples disease and potential malignancies were ruled out.

T2-FLAIR MRI showed hyperintensity involving the same region of midbrain, but less extensive than the one observed in the first episode, suggesting that the patient had a recurrent RE. The patient was again positive for ACA. The rest of the exams and tests were essentially normal. Given the negative infectious work up in the prior episode and the nearly identical clinical presentations, we decided to treat the patient with IV methylprednisolone 500 mg. This fully reversed her clinical symptoms and MRI lesions with no serious adverse events. At ten-month follow-up, routine investigations and examinations were normal with no residual neurological deficits. However, immunologic remission of ACA antibody was never achieved. Given the responsiveness to initial therapy, further treatments in chronic settings were not necessary. Plasma exchange and monoclonal antibody were not used due to the absence of severe symptoms, lack of other immunologic comorbidity, and unclear etiology.

## Discussion and conclusions

Rhombencephalitis (RE) affects individuals of all age**s**, sex, ethnicity, and immune status [4, 8, 9]. *Listeria monocytogenes* and other infectious inflammatory causes are often cited as the most common etiology of RE [10, 11]. However, some studies have reported a higher proportion of immunological causes among RE cohorts [4, 8]. PRES and anti-MOG RE in the differential diagnosis were excluded based on normal blood pressure, uncomplicated pregnancy, and negative AQP-4 antibodies. Rather, we focused on specific autoimmune RE and its autoantibodies, which may affect the brain in a specific way. Common initial findings for Anti-NMDAR RE include psychosis, memory impairment, dysmetria, and/or seizures [12]. Anti-Hu RE patients usually present with multifocal involvement of the brain that includes cerebellum and medulla, leading to cranial nerve and motor abnormalities [13]. Identifying specific antibody associated with RE may facilitate diagnosis, treatment, and prognosis [6].

Our patient was ultimately diagnosed with a primary autoimmune RE associated with ACA after excluding potential causes, such as autoimmune disease or paraneoplastic syndromes [2, 7–9, 14, 15]. MRI revealed a form of RE specifically affecting the midbrain of the brainstem. Interestingly, there seemed to be an association between her pregnancies and disease onset. The first episode of the autoimmune RE observed for this patient occurred during the postpartum period following the first pregnancy; and the same inflammatory process came back following the second pregnancy/delivery. Some cases of postpartum onset have been reported for anti-NMDAR encephalitis [16]. The underlying mechanism is not clear. This case extends the list of primary RE that occurs during the postpartum period.

It is intriguing to speculate about the timing of the recurrent RE, as well as the positive ACA. To the best of our knowledge, this is the first case of primary autoimmune RE associated with ACA. Previous studies have linked ANA to primary autoimmune RE with unknown etiology [4]. ACA is found in 13.4% of individuals with Sjögrens [17] and 50–96% with limited systemic scleroderma, also known as CREST syndrome [18]. Acute disseminated encephalopathy was reported in a patient with ACA-positive Sjögren that affected bilateral cerebral hemispheres, mainly within the white matter [19]. Even though it is rare, scleroderma may present with neurological manifestations involving the brainstem; and in that case it might mimic RE [20]. In our case, the patient did not meet the diagnostic criteria for Sjögrens nor scleroderma. This is therefore a new case of primary autoimmune RE associated with ACA.

ACA-associated RE seems to represent a unique subset of primary autoimmune RE that has autoantibodies with unknown effects on neurons. Other similar examples include anti-SSA and anti-MOG associated with Sjögrens and demyelinating diseases respectively [7, 21]. The precise role and prognostic value of ACA in the development and progression of autoimmune RE are unclear. There is no data to guide long-term treatment. Due to the potential for recurrence, relapse, and worsening degree of neurological symptoms in primary autoimmune RE, however, close monitoring of the patient is warranted, especially among women of child bearing age. Having a positive family history of SLE in conjunction with ACA, the patient may also have a higher probability of developing a connective tissue disease and thus may benefit from a long-term follow-up.

In conclusion, this case study reports an unusual case of recurrent postpartum, primary autoimmune RE in association with a positive ACA. We speculate there may be a causal association. In general, the diagnosis necessitates antibody testing of CSF, brain imaging, and exclusion of other causes of encephalitis, including infection, autoimmune disorders, drugs, and malignancy. Immunologic causes should be considered early with any encephalitis, given the risk of rapid deterioration and highly effective steroid treatment. A positive ACA status in the context of RE may guide acute treatment and long-term management. This paper supports the current consensus available for autoimmune RE - to use corticosteroid as the first-line therapy for ACA-associated RE. Further investigations on the functions of ACA in neurons are needed to better understand the pathophysiology.

## Data Availability

All data generated or analyzed during this study are included in this published article.

## Abbreviations

ACA: Anti-centromere antibody
ANA: Anti-nuclear antibody
AQP-4: Aquaporin-4
CSF: Cerebrospinal fluid
dsDNA: Double stranded deoxyribonucleic acid
FLAIR: Fluid attenuated inversion recovery
JO-1: Histidyl-tRNA synthetase antibody
MOG: Myelin oligodendrocyte glycoprotein
MRI: Magnetic resonance imaging
NMDAR: N-methyl-D-aspartate receptor
PRES: Posterior reversible encephalopathy syndrome
RE: Rhombencephalitis
RNP: Ribonucleoprotein
Scl-70: Topoisomerase I antibody
SLE: Systemic lupus erythematous
SSA: Sjögren’s syndrome related antigen A
SSB: Sjögren’s syndrome related antigen B
